# Recent advances in understanding and managing pediatric rhabdomyosarcoma

**DOI:** 10.12688/f1000research.22451.1

**Published:** 2020-07-08

**Authors:** Jessica Gartrell, Alberto Pappo

**Affiliations:** 1 Department of Oncology, St. Jude Children's Research Hospital, Memphis, TN, USA

**Keywords:** rhabdomyosarcoma, pediatric, sarcoma

## Abstract

Rhabdomyosarcoma (RMS) is a high-grade malignant neoplasm, with a morphologic appearance mimicking that of developing skeletal muscle. Over the last 30 years, patient outcomes have improved with the incorporation of multimodal therapies, including chemotherapy, radiation therapy, and surgery. The overall cure rates exceed 70%, with patients who have low-, intermediate-, and high-risk disease experiencing long-term survival rates of >90%, 70%, and <30%, respectively. Historically, RMS was classified according to histology; however, recent advances have revealed new molecular subgroups that allow us to more accurately identify high-, intermediate-, and low-risk disease. In this review, we discuss recent advances made in understanding RMS tumor biology and propose how this understanding can drive a new classification system that can guide clinical approaches for treatment de-escalation in patients with expected favorable outcomes and escalation for those with expected poor outcomes.

## Introduction

Rhabdomyosarcoma (RMS) is a high-grade malignant neoplasm, with a morphologic appearance mimicking that of developing skeletal muscle. It is the most common soft tissue sarcoma of childhood, accounting for 3% of all pediatric cancers ^1, 2^. Currently, the overall cure rates exceed 70%, with patients who have low-, intermediate-, and high-risk disease experiencing long-term survival rates of >90%, 70%, and <30%, respectively ^2– 4^. Multidisciplinary care and multimodal therapies, such as chemotherapy, radiation therapy, and surgery, have led to improvements in outcomes; however, new therapies are lacking and integrative computational analyses, functional approaches, and new molecular targets will be needed to further improve the outcome of this disease. Here, we summarize the most recent advances in the biology and treatment of RMS.

## Epidemiology

RMS has an overall incidence of 4.4 cases per 1 million in individuals under the age of 20 years ^1^. Predisposing factors for the development of RMS have included germline variations ( *DICER1* [urogenital and cervical embryonal RMS (eRMS)] ^5– 7^, Li-Fraumeni syndrome, Beckwith-Wiedemann syndrome, and RASopathies) ^8^, environmental factors such as prenatal X-ray exposure and maternal drug use, male sex, and age ^1, 3, 9^.

In a population-based study, Lupo *et al.* ^10^ identified familial and perinatal risk factors associated with developing soft tissue sarcomas. In an analysis of over 4 million individuals from Sweden, preterm birth was associated with an increased risk of RMS, implicating developmental pathways in the pathogenesis of the disease ^10^. In another study, Williams *et al.* ^11^ conducted a case-controlled study of 12,632 cases of childhood cancer from population-based cancer registries in Minnesota, New York, and Washington to determine the role of male sex on RMS oncogenesis. Male sex was directly associated with RMS, suggesting that sex-specific factors in gene expression patterns are responsible for the differences in the risk of developing childhood cancers.

## Pathology

The World Health Organization Classification of Soft Tissue and Bone identifies four prognostically different subtypes of RMS: eRMS, alveolar RMS (aRMS), pleomorphic, and spindle cell/sclerosing ^12– 14^. The two most common subtypes are eRMS and aRMS, in which eRMS accounts for 38.8% of cases and aRMS accounts for 22.3% of cases ^1^. The Children’s Oncology Group (COG) reviewed 2,192 patients enrolled in nine consecutive clinical trials to determine whether this classification system is valid for pediatric patients. They found that the system was generally valid for pediatric cohorts. Exceptions included low-risk botryoid tumors, which had an excellent prognosis, and spindle cell parameningeal tumors, which had a poor prognosis. The spindle cell/sclerosing group otherwise had a similar prognosis to eRMS, in contrast with adult data, which show that this histologic group conferred a poor prognosis. This study did not include molecular classification in the analyses, which may explain the differences between pediatric and adult data ^15^.

The aRMS subtype is characterized by PAX3–FOXO1 and PAX7–FOXO1 protein fusions. The COG retrospectively analyzed 1,727 patients treated in six of their most recent clinical trials. Their report found that fusion status was the most important prognostic factor for patients with localized and metastatic disease. The 5-year event free survival (EFS) rate was 52% for fusion-positive, localized disease; 6% for fusion-positive, metastatic disease; 78% for fusion-negative, localized disease; and 46% for fusion-negative metastatic disease ^16, 17^. The higher 5-year EFS rates for fusion-negative aRMS suggest that this group may unnecessarily receive therapies for high-risk disease, as these outcomes are similar to those of eRMS cases treated with intermediate-risk therapies. Therefore, this represents a group of patients in whom therapy de-escalation should occur ^13, 16, 18, 19^.

New molecular subtypes of RMS include *MYOD1*-mutant RMS, *VGLL2/NCOA2*-rearranged RMS, and *TFCP2*-rearranged RMS. Identification of these subtypes has clinical relevance and may allow further stratification of patients in future clinical trials. For example, patients with the spindle cell subtype who are younger than 5 years of age frequently harbor either a *VGLL2* or a *NCOA2* fusion. These tumors arise nearly exclusively in soft tissues, and small case reports have shown that these patients have excellent clinical outcomes ^13, 20^. Butel *et al.* ^21^ reviewed the clinical and molecular findings of 37 infants with RMS (<6 months of age). They reported 22% having the spindle cell subtype and described two separate histologic pictures. Tumors with *VGLL2/NCOA2* fusions presented with a histology that was more “fibromatous-like” versus those with a “fibrosarcoma-like” picture, which had rearrangements in *TPM3–NTRK1*, *SYPL1–BRAF*, and *TOP2B–RAF1*. Both subtypes had excellent clinical outcomes. Thus, this group may be particularly favorable for the evaluation of dose de-escalation, or modifications in local control measures may be warranted.

The *MYOD1* mutations in the spindle cell/sclerosing subtype are found more frequently in older patients ^9, 13^. Agaram *et al.* ^9^ retrospectively investigated 30 cases of *MYOD1* mutant RMS, of which 15 were pediatric cases. The clinical outcomes for these patients were poor, with a 4-year survival rate of only 18% and 15 of 22 (68%) dying of disease between 12 and 68 months after diagnosis. Approximately one-third of the cases had a concomitant *PIK3CA* mutation, potentially providing a targeted therapy for these patients. RAS pathway mutations occur in 50 to 75% of intermediate- and high-risk RMS cases ^18, 22^. Although combination therapy with CDK4/6 and MEK inhibitors is an apparent rational combination, *in vivo* studies ^18^ failed to show activity, suggesting that different combination strategies should be explored.

## Genetics

Casey *et al.* ^23^ ** identified 87 patients with RMS who had undergone genomic profiling with a 468-gene onco-panel. Patients with fusion-negative RMS (n = 65) had more genomic alterations and higher tumor mutational burdens (TMBs) than patients with fusion-positive RMS did. The most common genomic abnormalities in patients with fusion-negative RMS were mutations in *TP53*, *NF1*, *MYOD1*, *NRAS*, ** and *BCOR*; ** deletions in *CDKN2*; ** and amplifications in *CDK4*, *MDM2*, ** and *GLI.* In contrast, fusion-positive tumors had “quiet” genomes, with *CDK4* amplifications as the only notable genetic alterations. This study also showed that high TMB was associated with poor clinical outcomes, regardless of fusion status, stage, or treatment, suggesting that this marker could be explored further as a way of stratifying patients in the future ^23^.

## Risk stratification

The current staging systems for RMS include histology, post-surgical status, tumor location, nodal involvement, tumor size, patient age, and tumor stage. The European Paediatric Soft Tissue Sarcoma Study Group (EpSSG) RMS 2005 trial defined four different risk groups: low, standard, high, and very high risk ( Table 1). In contrast, the COG identifies only three risk groups: low, intermediate, and high ( Table 2). The most notable difference in these stratification systems is how the two organizations stratify patients with aRMS. Those treated in EpSSG trials are considered high risk, and those with nodal involvement are considered very high risk ^24, 25^. In contrast, patients with non-metastatic aRMS are considered intermediate risk in the current COG stratification system. These subtle differences make comparisons of outcomes-based risk assessments problematic and highlight the need for collaborative efforts to agree upon a global definition of risk group.

**Table 1.** Risk stratification and treatment based on European Paediatric Soft Tissue Sarcoma Study Group completed trials.

Risk	Histology	Age (years)	Nodal involvement	Tumor site	Tumor size (cm)	IRS group	Treatment
Low	eRMS	<10	N0	Any	≤5	I	VA × 22 weeks
Standard	eRMS	≥10	N0	Favorable	>5	I	IVA × 12 weeks + VA 14 weeks NO RT
	eRMS	Any	N0	Favorable	Any	II–III	IVA × 9 weeks + (IVA × 4 weeks + VA × 8 weeks + RT) OR (IVA × 12 weeks + NO RT)
	eRMS	<10	N0	Unfavorable	≤5	II–III	IVA × 27 weeks + RT
High	eRMS	≥10	N0	Unfavorable	>5	II–III	IVA × 27 weeks + (vinorelbine + cyclophosphamide) × 24 weeks
	eRMS	<10	N1	Favorable	≤5	I–III	IVA × 27 weeks + (vinorelbine + cyclophosphamide) × 24 weeks
	aRMS	Any	N0	Any	Any	Any	IVA × 27 weeks + (vinorelbine + cyclophosphamide) × 24 weeks
Very high risk	aRMS	Any	N1	Any	Any	Any	IVADo × 12 weeks + IVA × 15 weeks + (vinorelbine + cyclophosphamide) × 24 weeks

aRMS, alveolar rhabdomyosarcoma; eRMS, embryonal rhabdomyosarcoma; IVA, ifosfamide, vincristine, and actinomycin D; IVADo, ifosfamide, vincristine, actinomycin D, and doxorubicin; IRS, Intergroup Rhabdomyosarcoma Studies; RT, radiation therapy; VA, vincristine and actinomycin D.

**Table 2.** Risk stratification and treatment based on Children’s Oncology Group completed and ongoing trials.

Risk	Protocol	Histology	Fusion status	TNM stage	IRS group	Treatment
Low risk	ARST0331	eRMS	n/a	1	I–II	VAC × 12 weeks + VA × 12 weeks
				1	III (orbital)	
				2	I–II	
Intermediate	ARST1431 (ongoing)	eRMS	n/a	1	III (non- orbital)	VAC/VI +/– temsirolimus × 42 weeks + cyclophosphamide + vinorelbine × 23 weeks
		eRMS	n/a	3	I–II	VAC/VI +/– temsirolimus × 42 weeks + cyclophosphamide + vinorelbine × 23 weeks
		eRMS	n/a	2–3	III	VAC/VI +/– temsirolimus × 42 weeks + cyclophosphamide + vinorelbine × 23 weeks
		eRMS	n/a	4 (age <10 years)	IV (age <10 years)	VAC/VI +/– temsirolimus × 42 weeks + cyclophosphamide + vinorelbine × 23 weeks
		aRMS	+	1–3	I–III	VAC/VI +/– temsirolimus × 42 weeks + cyclophosphamide + vinorelbine × 23 weeks
		aRMS	–	1–2	I–II + III (orbit)	VAC × 12 weeks + VA × 12 weeks
High risk	ARST0431	eRMS	n/a	4	IV (age ≥10 years)	VDC/IfosE + VI × 54 weeks
		aRMS	+/–	4	IV	

aRMS, alveolar rhabdomyosarcoma; eRMS, embryonal rhabdomyosarcoma; IfosE, ifosfamide and etoposide; IRS, Intergroup Rhabdomyosarcoma Studies; n/a, not applicable; TNM, tumor, node, and metastasis; VA, vincristine and actinomycin D; VAC, vincristine, actinomycin D, and cyclophosphamide; VDC, vincristine, doxorubicin, and cyclophosphamide; VI, vincristine and irinotecan.

More recently, the COG has incorporated fusion status into their risk stratification system. For example, in the current COG ARST1431 trial (NCT02567435), patients with fusion-negative stage 1, group I/II; stage 1, group III orbital; or stage 2, group I/II disease are treated with a similar strategy as that for patients with low-risk disease. Additional efforts should be made to incorporate novel subtypes, such as those with *MYOD1* mutations, increased TMB, VGLL2 and NCOA2 protein fusions, or *TP53* mutations, into risk-stratification systems for clinical trials ^9, 13, 20^.

## Advances in the treatment of RMS

Using the COG’s current risk group classification ( Table 2), we describe the most recent treatment advances in these subsets.

### Low-risk disease

Patients with low-risk RMS have survival rates exceeding 90%; therefore, this population is appropriate for studies with less-intense therapies to limit acute and long-term toxicities ^26^. The COG ARST0331 (NCT00075582) trial was designed to decrease exposure to alkylating agents and reduce the duration of therapy in selected groups of patients with low-risk disease. Patients with stage 1–2, group I–II or with stage 1, group III orbital eRMS tumors were treated with 22 weeks of therapy. The treatment regimen comprised four cycles of vincristine, actinomycin D, and cyclophosphamide (VAC), with a total cyclophosphamide cumulative dose of 4.8 g/m ^2^, followed by four cycles of vincristine and actinomycin D. In addition, radiation doses were decreased from 41.4 Gy to 36 Gy for patients with group IIA and from 50.4 Gy to 45 Gy for those with orbital group III eRMS tumors. Over a 6-year period, 271 patients were enrolled in the trial. The most common primary tumor sites were paratesticular (n = 118) and orbital (n = 82). The 3-year failure-free survival (FFS) and overall survival (OS) rates were 89% and 98%, respectively. For specific sites, the 3-year FFS and OS rates were 93% and 99% for paratesticular tumors, 86% and 97% for orbital tumors, and 86% and 97% for all other patients, respectively ^26^. Patients with orbital tumors who were treated with 45 Gy of radiation and did not achieve a complete response after 12 weeks of chemotherapy had higher local failure rates, but the OS rate was similar between the two groups ^27^. In contrast to these excellent results, patients with embryonal stage 1 group III non-orbital tumors or stage 3 group I/II were treated with four VAC cycles and 12 cycles of vincristine and actinomycin D (VA) and experienced a lower FFS of 70% and OS of 92%, mainly owing to local failures in patients with genital tract tumors ^28^. Therefore, this group of patients are now considered intermediate risk.

The EpSSG is evaluating therapy for patients with low-risk RMS by eliminating cyclophosphamide from therapy for patients who are younger than 10 years old and have a group I eRMS tumor that is <5 cm with no nodal involvement. The results of this trial will inform future therapies for pediatric patients with low-risk RMS.

### Intermediate-risk disease

Over 50% of patients with RMS have intermediate-risk disease. These patients have either nonmetastatic eRMS with an unfavorable primary site or nonmetastatic aRMS. The OS rate for this group approaches 75% ^4^, with no marked improvements occurring in the last few decades. The COG developed a randomized study (ARST0531; NCT00354835) to improve outcomes in this group by incorporating vincristine and irinotecan (VI) into the VAC backbone. The rationale for incorporating VI was based on the very high rates of response (70%) occurring in patients with high-risk RMS who were treated with two courses of VI as a window therapy ^29, 30^. The ARST0531 trial randomized 448 patients to receive either VAC or VAC/VI, in which VI replaced VAC in weeks 16, 19, 25, 31, and 37 ^30^. The trial design resulted in a 45% dose reduction of cyclophosphamide in the VAC group and a 66% reduction of cyclophosphamide in the VAC/VI group as compared with a previous intermediate-risk group trial (D9803; NCT00003958) ^31^. The two arms had similar outcomes, with a 4-year EFS rate of 63% in the VAC group and 59% in the VAC/VI group ( *P* = 0.51). Despite a lack of improvement between the two groups, the VAC/VI group had lower rates of anemia, thrombocytopenia, and febrile neutropenia but higher rates of diarrhea and mucositis ^30^.

Further analysis of this trial demonstrated that the rates of local failure were higher than those of the D9803 trial. The local failure rates for patients with tumors >5 cm were 32.3% versus 16.7% ( *P* = 0.001) and 27.9% versus 19.4% for those with group III eRMS ( *P* = 0.03) ^32^. In addition, the EFS and OS rates for patients in the ARST0531 trial were lower than those for patients in the D9803 trial. These differences were most apparent for patients with stage 2/3, group III eRMS tumors, comprising 54% of the patients enrolled in the ARST0531 trial. The reasons for this difference are not entirely clear but may be related to lower cumulative doses of cyclophosphamide ^31, 33, 34^. Increased failure rates have been observed in patients with head and neck RMS treated with lower doses of cyclophosphamide ^31, 33, 34^, and improvements in outcomes were found in the EpSSG RMS 2005 study for patients treated with higher doses of cyclophosphamide ^24, 31^. This supports the notion that there may be a minimal dose threshold of alkylating agent required with current treatment strategies.

The EpSSG RMS 2005 trial randomized patients with high-risk RMS (patients with nonmetastatic, incompletely resected RMS, eRMS occurring at unfavorable sites, age 10 years or older or with a tumor size of >5 cm or both, and any nonmetastatic eRMS with nodal involvement), who were stratified in a similar way to that of intermediate-risk RMS in the COG trial ( Table 2), into two consecutive independent randomized trials. In the first trial, 484 patients were randomized to receive nine cycles of ifosfamide, vincristine, and actinomycin D (IVA) or four cycles of IVA and doxorubicin (IVADo) followed by five cycles of IVA. Patients who were in remission following upfront therapy were randomized on the second trial to either stop treatment or receive a maintenance regimen with low-dose cyclophosphamide and vinorelbine. The addition of doxorubicin only increased toxicity without improving survival, with a 3-year EFS of 67.5% in the IVADo group and 63.3% in the IVA group ^25^. However, the addition of maintenance therapy in the second trial improved disease-free survival (77.6% with maintenance versus 69.8% without) and OS (86.5% with maintenance versus 73.7% without) ^24^. Of the initial 670 patients assessed for eligibility for the maintenance trial, 12.1% were excluded because they did not achieve remission at the end of standard of treatment ^24^. Additional follow-up is needed to determine the impact of maintenance therapy on long-term survival.

On the basis of the improvements found in the EpSSG RMS 2005 maintenance trial, the COG’s current trial for intermediate-risk disease (ARST1431; NCT02567435) will use the same VAC/VI backbone used in ARST0531 but adopt the maintenance approach with vinorelbine and cyclophosphamide, increasing the total cumulative cyclophosphamide dose from 8.4 g/m ^2^ to 12.6 g/m ^2^. The importance of duration versus total cumulative dose of cyclophosphamide is not yet known.

### High-risk disease

Historically, the outcomes for patients with metastatic RMS are poor, with <30% surviving long term. The COG ARST0431 trial enrolled 109 patients with metastatic disease to receive 54 weeks of therapy, consisting of VI (weeks 1–6, 20–25, and 47–52) with interval compressed vincristine/doxorubicin/cyclophosphamide alternating with etoposide/ifosfamide (weeks 7–19 and 26–34) and then VAC (weeks 38–46). They found that the number of Oberlin risk factors—age >10 years or <1 year, unfavorable primary site of disease, three or more metastatic sites, and bone or bone marrow involvement—had prognostic significance. The patients with no or one Oberlin risk factor (n = 43) had improved outcomes over those of historical control cohorts. The EFS was 67% in the ARST0431 trial and 44% in the Oberlin-controlled cohort; however, those with two or more risk factors experienced poor outcomes, with an EFS of 19% in the ARST0431 trial and 14% in the Oberlin-controlled cohort. Furthermore, when the patients were stratified by histology, only those with eRMS experienced benefits from this treatment regimen ^35^.

Another recently completed COG non-randomized trial (ARST08P1; NCT01055314) explored the addition of either an insulin-like growth factor-1 receptor monoclonal antibody, cixutumumab, or temozolomide to the ARST0431 trial backbone. There were 168 patients enrolled, 70% of whom had aRMS. Patients who were <10 years old with eRMS were initially excluded until safety was determined. With a median follow-up of 2.9 years, the 3-year EFS was 16% (95% confidence interval [CI]: 7–25%) with cixutumumab and 18% (95% CI: 2–35%) with temozolomide. Both arms had an inferior EFS compared to the EFS of 38% achieved in ARST0431. This may be due in part to the early exclusion of the young eRMS cohort. Consistent with ARST0431, Oberlin risk factors continued to be prognostic, with patients with fewer than two Oberlin risk factors having a 3-year EFS of 38% compared to an EFS of 9% for those with two or more Oberlin risk factors. For both Oberlin risk groups, the EFS was inferior to those patients treated on ARST0431 ^36^.

The EpSSG BERNIE study randomized 103 patients with metastatic RMS and 49 patients with metastatic non-RMS soft tissue sarcoma to receive either IVADo alone or in combination with the VEGF inhibitor bevacizumab, followed by maintenance chemotherapy with cyclophosphamide and vinorelbine with or without bevacizumab. The hazard rate for EFS in patients with RMS was 1.24 (95% CI: 0.73–2.09). The independent review committee (IRC) response rate for aRMS was 64% with bevacizumab (95% CI: 42.5–82) and 53.1% (95% CI: 34.7–70.9) without bevacizumab. For eRMS, the IRC response rate was 66.7% with bevacizumab (95% CI: 41–86.7) and 53.3% (95% CI: 26.6–78.7) without bevacizumab. The 2-year EFS remained 41% for both treatment groups ^37^. Therefore, it is clear that new treatment strategies are needed for patients with metastatic RMS.

### Recurrent/refractory disease

Most patients with recurrent/refractory disease experience poor clinical outcomes. To determine whether delayed diagnosis contributes to such poor outcomes in some patients, the EpSSG conducted a retrospective study comparing 78 patients who had imaging performed routinely to 121 patients who had imaging performed on the basis of symptoms alone. The patients who received routine imaging had their disease detected earlier than those who did not (8 versus 12 months); however, this did not improve survival, suggesting that the poor outcomes cannot be explained solely by delayed diagnosis ^38^.

The Italian Soft Tissue Sarcoma Committee treated 38 patients with progressive or recurrent disease with two cycles of topotecan and carboplatin, followed by four cycles of topotecan and cyclophosphamide and alternating with carboplatin and etoposide. Of 38 patients, 32 completed the therapy with an overall response rate of 37.5% (35% for aRMS versus 20% for eRMS). Nevertheless, this did not improve OS, which remained at 17% ^39^.

The COG study ARST0121 enrolled 139 patients experiencing first recurrence/relapse and divided them into four treatment groups based on risk groups. Those with “unfavorable risk factors” (initial stage 2–4, clinical group II–IV eRMS, initial stage I or clinical group I eRMS with distant recurrence after VA or recurrence after VAC, and aRMS) were randomized to receive differing schedules of window therapy with VI followed by dose-intensive chemotherapy with vincristine/doxorubicin/cyclophosphamide/ifosfamide/etoposide/irinotecan. If they refused window therapy, or had prior irinotecan exposure, window therapy and irinotecan were omitted and a new agent, tirapazamine (TPZ), was added. Alternatively, those with “favorable risk” disease (botryoid histology, or stage 1, or locally or regionally recurring clinical group I eRMS at initial diagnosis not treated with cyclophosphamide) received the chemotherapy regimen without window therapy, irinotecan, or TPZ. While patients with unfavorable risk continued to have poor outcomes (FFS 14% and 15% with window therapy regimens, 21% with TPZ), the study confirmed that those with favorable risk had good outcomes (FFS 79%, OS 84%) ^40^.

The COG study ARST0921 (NCT01222715) randomized 87 patients with a first recurrence to receive either bevacizumab or temsirolimus in combination with vinorelbine and cyclophosphamide. The patients who received temsirolimus had a superior EFS than did those who received bevacizumab. The 6-month EFS was 69.1% for patients who received temsirolimus and 54.6% for those who received bevacizumab; however, the 24-month EFS was less than 20% for both groups, with 19.1% for temsirolimus and 6.8% for bevacizumab ^41^.

To identify new targeted therapies, Stewart *et al.* used orthotopic patient-derived xenograft models to perform an integrated transcriptomic, epigenetic, and proteomic/phosphoproteomic analysis of refractory RMS tumors and identified significant activity with the WEE1 inhibitor AZD1775 in combination with vincristine and irinotecan (NCT02095132) ^42^.

Yan *et al.* ^43^ used immunodeficient zebrafish to successfully engraft both eRMS and aRMS patient-derived xenografts, offering an opportunity to expand preclinical testing in this disease. They identified that the combination of olaparib with temozolomide was active and validated these findings in cell lines and patient-derived xenograft mouse models.

## Conclusion

Despite advances in the cellular and genomic classification of RMS and the introduction of combined modality therapy, outcomes for patients at high risk for treatment failure remain suboptimal. In addition, patients who survive their disease are at high risk of developing long-term sequelae as a result of systemic and local therapies. Additional progress will require the development of accurate models that recapitulate human disease, integration of genomics in risk classification, biomarker development, and better informatics that allow for the creation of a data commons to seamlessly integrate data to catalyze discovery.
